# A Point-based Mortality Prediction System for Older Adults with Diabetes

**DOI:** 10.1038/s41598-017-12751-3

**Published:** 2017-10-04

**Authors:** Y. K. Chang, L. F. Huang, S. J. Shin, K. D. Lin, K. Chong, F. S. Yen, H. Y. Chang, S. Y. Chuang, T. J. Hsieh, C. A. Hsiung, C. C. Hsu

**Affiliations:** 1 Department of Medical Research, Tung’s Taichung MetroHarbor Hospital, Taichung, Taiwan; 2 Institute of Population Health Sciences, National Health Research Institutes, Zhunan, Taiwan; 3 College of Medicine, Kaohsiung Medical University, Kaohsiung, Taiwan; 4 Ditvision of Endocrinology and Metabolism, Department of Internal Medicine, Kaohsiung Medical University Hospital, Kaohsiung, Taiwan; 5 Department of Internal Medicine, Kaohsiung Municipal Ta-Tung Hospital, Kaohsiung, Taiwan; 6 Division of Endocrinology and Metabolism, Department of Internal Medicine, Min-Sheng General Hospital, Taoyuan, Taiwan; 7 Dr. Yen’s Clinic, Taoyuan, Taiwan; 8 Department of Health Services Administration, China Medical University, Taichung, Taiwan; 9 Department of Family Medicine, Min-Sheng General Hospital, Taoyuan, Taiwan

## Abstract

The mortality prediction models for the general diabetic population have been well established, but the corresponding elderly-specific model is still lacking. This study aims to develop a mortality prediction model for the elderly with diabetes. The data used for model establishment were derived from the nationwide adult health screening program in Taiwan in 2007–2010, from which we applied a 10-fold cross-validation method for model construction and internal validation. The external validation was tested on the MJ health screening database collected in 2004–2007. Multivariable Cox proportional hazards models were used to predict five-year mortality for diabetic patients ≥65 years. A total of 220,832 older subjects with diabetes were selected for model construction, of whom 23,241 (10.5%) died by the end of follow-up (December 31, 2011). The significant predictors retained in the final model included age, gender, smoking status, body mass index (BMI), fasting glucose, systolic and diastolic blood pressure, leukocyte count, liver and renal function, total cholesterol, hemoglobin, albumin, and uric acid. The Harrell’s C in the development, internal-, and external-validation datasets were 0.737, 0.746, and 0.685, respectively. We established an easy-to-use point-based model that could accurately predict five-year mortality risk in older adults with diabetes.

## Introduction

Population aging is the most important mainspring of the escalating growth of diabetes prevalence throughout the world^1^. According to the report from the US Centers for Disease Control and Prevention, the prevalence of diabetes in older Americans (≥65 years) was 25.9% of in 2012^2^, which was much higher than those at age 20–44 (4.1%) and 45–64 (16.2%). Similarly, aging has had a significant impact on the rise of diabetes in Asian populations^3^. In addition to being susceptible to micro- and macro-vascular complications, older adults with diabetes are prone to develop premature death or hypoglycemia^4,5^. The Emerging Risk Factors Collaboration disclosed that the elderly people with diabetes were associated with substantial premature mortality from vascular, cancers, renal, liver, digestive diseases and infection which incurred about 4–5 years of life loss^6^. However, this vulnerable group receives less attention by empirical studies. In renowned guidelines, most healthcare standards proposed for older adults with diabetes were based on expert consensus^7^. Furthermore, some recommended goals for blood pressure or glycemic control derived from large-scale randomized control trials^8,9^ may be inappropriate to apply to older patients, who often suffer from physically dysfunction or multiple comorbidities. To improve diabetes care for the elderly, we are in urgent need of developing an evidence-based clinical guidance for risk stratification and therapeutic purpose. In recent years, some prediction models have been separately established to estimate the mortality risk for older people^10–12^ and diabetic patients^13–15^. Moreover, a one-year mortality prediction model was constructed for diabetic patients with dialysis commencement^16^. To our knowledge, there is still lack of a prediction model that can identify high-risk groups and can prevent premature mortality for an older population with diabetes. Therefore, we incorporated factors that may influence health outcomes for these patients, including glucose and blood pressure control, metabolic syndrome, renal and liver function, and nutritional and inflammatory status, to form a thorough set of predictors and then developed a reliable and validated risk score system to predict five-year mortality of diabetic patients age 65 or older.

## Results

Table 1 shows the demographic characteristics as well as the clinical and biochemical measures of the older subjects with diabetes in the development, internal-, and external-validation datasets. There were no significant differences between subjects in the internal-validation and development datasets. Compared to the subjects in the development dataset, those in the external-validation dataset were younger, more likely to be male, more likely to smoke, and had higher levels of the biochemical measures such as fasting blood glucose, systolic blood pressure (SBP), total cholesterol (TCHOL), albumin (Alb), hemoglobin (Hb), estimated glomerular filtration rate (eGFR), glutamic pyruvic transaminase (GPT), and uric acid (UA).

**Table 1:** Baseline characteristics of the older subjects with diabetes.

	Development dataset	Validation datasets			
	AHSP data (n = 220,832)	AHSP data for internal validation (n = 24,538)		MJ data for external validation (n = 2,093)	
			p value^a^		p value^b^
Men (%)	45.32	45.49	0.613	50.41	<0.001
Age (years)	74.1 ± 6.4	74.3 ± 6.5	0.012	70.2 ± 4.9	<0.001
Ever smoker (%)	8.08	7.76	0.083	24.80	<0.001
Follow-up years	3.0 ± 1.2	3.0 ± 1.2	0.826	3.2 ± 1.2	<0.001
BMI (kg/m^2^)	25.2 ± 3.7	25.1 ± 3.7	0.552	25.0 ± 3.3	0.026
Fasting glucose (mg/dl)^c^	151.2 ± 56.3	150.6 ± 55.9	0.125	155.3 ± 47.7	<0.001
SBP (mmHg)	137.8 ± 18.7	138.0 ± 18.8	0.164	139.∙4 ± 19.7	<0.001
DBP (mmHg)	78.7 ± 10.7	78.7 ± 10.7	0.608	75.1 ± 11.3	<0.001
TCHOL (mg/dl)^d^	194.7 ± 41.6	194.7 ± 41.5	0.963	200.2 ± 38.9	<0.001
TG (mg/dl)^e^	159.0 ± 111.3	157.9 ± 109.0	0.106	160.1 ± 100.4	0.671
Hb (g/dl)^f^	13.4 ± 1.7	13.4 ± 1.7	0.481	14.1 ± 1.5	<0.001
Alb (g/dl)^g^	4.2 ± 0.4	4.2 ± 0.4	0.499	4.5 ± 0.3	<0.001
eGFR (mL/min/1.73 m^2^)	62.2 ± 19.6	62.1 ± 19.6	0.339	67.8 ± 16.3	<0.001
WBC (/μl)	6,750.4 ± 1,780.0	6,744.2 ± 1,776.6	0.603	6,599.9 ± 1,648.3	<0.001
GPT (U/l)	28.6 ± 23.8	28.9 ± 24.1	0.101	30.8 ± 23.3	<0.001
UA (mg/dl)^h^	6.0 ± 1.7	6.0 ± 1.7	0.867	6.1 ± 1.6	0.006

The data indicate mean ± sd or %. ^a^The chance to reject null hypotheses that there is no significant difference between subjects in development dataset and internal validation dataset. ^b^The chance to reject null hypotheses that there is no significant difference between subjects in development dataset and external validation dataset. ^c^mg/dl × 0.0555 = mmol/L (US units to SI units). ^d^mg/dl × 0.0259 = mmol/L (US units to SI units). ^e^mg/dl × 0.0113 = mmol/L (US units to SI units). ^f^mg/dl × 0.6206 = mmol/L (US units to SI units). ^g^g/dl × 10 = g/l (US units to SI units). ^h^mg/dl × 0.0168 = µmol/L (US units to SI units). AHSP, Adult Health Screening Program; MJ, MJ Health Screening Center; SBP, systolic blood pressure; DBP, diastolic blood pressure; TCHOL, total cholesterol; TG, triglyceride; Hb, hemoglobin; Alb, albumin; eGFR, estimated glomerular filtration rate; WBC, white blood cell count; GPT, glutamic pyruvic transaminase; UA, uric acid.

As shown in Table 2, the overall mortality rate of the subjects in the development and internal validation datasets were similar (3.38–3.48 deaths per 100 person-years), but higher than that in the external validation dataset (1.38 per 100 person-years). The lower mortality rates in the external validation dataset were even more obvious in males, and those older than 75.

**Table 2:** All-cause mortality rate of the older subjects with diabetes.

	Development dataset	Internal validation dataset	External validation dataset
Overall (N)	220,832	24,538	2,093
Death toll	23,241	2,515	91
Person-years followed up^a^	668,605.7	74,335.17	6,597.55
Mortality rate^b^	3.48	3.38 (3.25–3.51)	1.38 (1.10–1.66)
Male (n)	100,079	11,162	1,055
Death toll	12,854	1,392	54
Person-years followed up^a^	298,642.2	33,275	3,365.22
Mortality rate^b^	4.30	4.18 (3.97–4.40)	1.60 (1.18–2.03)
Female (n)	120,753	13,376	1,038
Death toll	10,387	1,123	37
Person-years followed up^a^	369,963.5	41,060.17	3,232.33
Mortality rate^b^	2.81	2.74 (2.58–2.89)	1.14 (0.80–1.51)
65–74 years old (n)	125,412	13,729	1,679
Death toll	7,398	736	59
Person-years followed up^a^	386,568.8	42,358.58	5,288.23
Mortality rate^b^	1.91	1.74 (1.61–1.86)	1.12 (0.83–1.40)
≥75 years old (n)	95,420	10,809	414
Death toll	15,843	1,779	32
Person-years followed up^a^	282,036.9	31,976.59	1,309.32
Mortality rate^b^	5.62 (5.53–5.70)	5.56 (5.31–5.82)	2.44 (1.60–3.28)

^a^The follow-up period was from enrollment to the end of the 5th year. ^b^Per 100 person-years-calculated by Jackknife mean (95% confidence interval was calculated by Jackknife resampling).

The estimated relative risks of mortality are shown in Table 3. The hazard ratio (HR) is 1.07 for every one-year increment in age. Males and ever smokers were more likely to die (HR = 1.70 and 1.13, respectively). Some measured biomarkers demonstrated a U-shaped effect on risk of death. For example, HR was greater than 1 for fasting blood glucose of being either <70 mg/dL or ≥140 mg/dL. Similar harmful effects could be seen at both extreme levels of TCHOL (<150 or ≥240 mg/dL), eGFR (<60 or ≥100 mL/min/1.73 m^2^), and UA (<3.5 or ≥7 mg/dL). The mortality risk would be increased for those with body mass index (BMI) < 24 kg/m^2^, SBP < 110 mmHg, diastolic blood pressure (DBP) ≥80 mmHg, Hb < 14 g/dL, Alb < 4 g/dL, white blood cell count (WBC) ≥ 8,200/µL, or GPT ≥ 40 U/L. If an older adult who has diabetes as well as albumin < 3.5 g/dL, the HR would be increased by 3.64 fold.

**Table 3:** Risk factors and the point system to predict five-year all-cause mortality for the older subjects with diabetes.

Risk factors	Estimated coefficient	Hazard ratio (95% CI)	p value	Point
Age^a^	0.07	1.07 (1.07–1.07)	<0.001	
65–69		Ref.		0
70–74				1
75–79				2
80–84				3
85–89				4
90–99				5
Male	0.53	1.70 (1.65–1.75)	<0.001	2
Ever smoker	0.12	1.13 (1.07–1.18)	<0.001	1
BMI (kg/m^2^)				
<18.5	0.78	2.19 (2.07–2.31)	<0.001	3
18.5–21.9	0.42	1.52 (1.47–1.58)	<0.001	2
22–23.9	0.20	1.22 (1.18–1.27)	<0.001	1
24–29.9		Ref.		0
≥30	0.03	1.03 (0.98–1.09)	0.253	0
Fasting glucose (mg/dl)^b^				
<70	0.29	1.34 (1.21–1.48)	<0.001	1
70–139		Ref.		0
140–159	0.12	1.13 (1.09–1.17)	<0.001	1
160–199	0.21	1.23 (1.19–1.28)	<0.001	1
≥200	0.37	1.44 (1.39–1.50)	<0.001	2
SBP (mmHg)				
<110	0.15	1.16 (1.09–1.23)	<0.001	1
110–119	0.03	1.04 (0.99–1.08)	0.132	0
120–159		Ref.		0
160–169	0.00	1.00 (0.95–1.06)	0.889	0
≥170	0.04	1.04 (0.99–1.10)	0.155	0
DBP (mmHg)				
<80		Ref.		0
80–89	0.08	1.08 (1.05–1.11)	<0.001	1
≥90	0.16	1.18 (1.13–1.22)	<0.001	1
TCHOL (mg/dl)^c^				
<140	0.17	1.18 (1.13–1.23)	<0.001	1
140–149	0.14	1.15 (1.09–1.21)	<0.001	1
150–239		Ref.		0
≥240	0.05	1.05 (1.00–1.09)	0.035	1
TG (mg/dl)^d^				
<120	−0.02	0.98 (0.94–1.03)	0.426	0
120–159	0.02	1.02 (0.97–1.07)	0.471	0
160–199		Ref.		0
200–239	−0.05	0.95 (0.90–1.01)	0.105	0
≥240	0.01	1.01 (0.96–1.07)	0.667	0
Hb (g/dl)^e^				
<12	0.71	2.03 (1.95–2.11)	<0.001	3
12–12.9	0.33	1.40 (1.34–1.46)	<0.001	1
13–13.9	0.19	1.21 (1.16–1.26)	<0.001	1
≥14		Ref.		0
Alb (g/dl)^f^				
<3.5	1.29	3.64 (3.49–3.80)	<0.001	4
3.5–3.9	0.66	1.93 (1.87–1.99)	<0.001	2
≥4.0		Ref.		0
eGFR (mL/min/1.73 m^2^)				
≥100	0.25	1.29 (1.11–1.50)	0.001	1
60–99		Ref.		0
45–59	0.07	1.07 (1.04–1.11)	<0.001	1
30–44	0.20	1.23 (1.18–1.27)	<0.001	1
<30	0.55	1.73 (1.65–1.81)	<0.001	2
WBC (/μL)				
<8,200		Ref.		0
8,200–9,999	0.26	1.29 (1.25–1.34)	<0.001	1
≥10,000	0.42	1.53 (1.46–1.60)	<0.001	2
GPT (U/L)				
<40		Ref.		0
40–79	0.08	1.08 (1.04–1.13)	<0.001	1
80–119	0.33	1.39 (1.28–1.51)	<0.001	1
≥120	0.42	1.52 (1.37–1.67)	<0.001	2
UA (mg/dl)^g^				
<3.5	0.08	1.09 (1.01–1.16)	0.019	1
3.5–6.9		Ref.		0
7–8.9	0.05	1.06 (1.02–1.09)	0.001	1
≥9	0.21	1.23 (1.17–1.29)	<0.001	1

Note: the hazards ratios were estimated by using the multivariable Cox proportional hazards model. ^a^The age was divided into six categories as shown in Table 3. The regression coefficient was assessed by treating age as a dummy variable (65–69: 0; 70–74: 1; 75–79: 2; 80–84: 3; 85–89: 4; 90–99: 5). For the point calculation, we added 1 to each higher rank of age categories. Then, we let 5 multiply the regression coefficient of age as a reference to transform the regression coefficient in each significant category of investigated covariates into a risk point estimate. Ref., reference group; SBP, systolic blood pressure; DBP, diastolic blood pressure; TCHOL, total cholesterol; TG, triglyceride; Hb: hemoglobin; Alb: albumin; eGFR, estimated glomerular filtration rate; WBC, white blood cell count; GPT, glutamic pyruvic transaminase; UA, uric acid. ^b^mg/dl × 0.0555 = mmol/L (US units to SI units). ^c^mg/dl × 0.0259 = mmol/L (US units to SI units). ^d^mg/dl × 0.0113 = mmol/L (US units to SI units). ^e^mg/dl × 0.6206 = mmol/L (US units to SI units). ^f^g/dl × 10 = g/l (US units to SI units). ^g^mg/dl × 0.0168 = µmol/L (US units to SI units).

**Figure 1:**
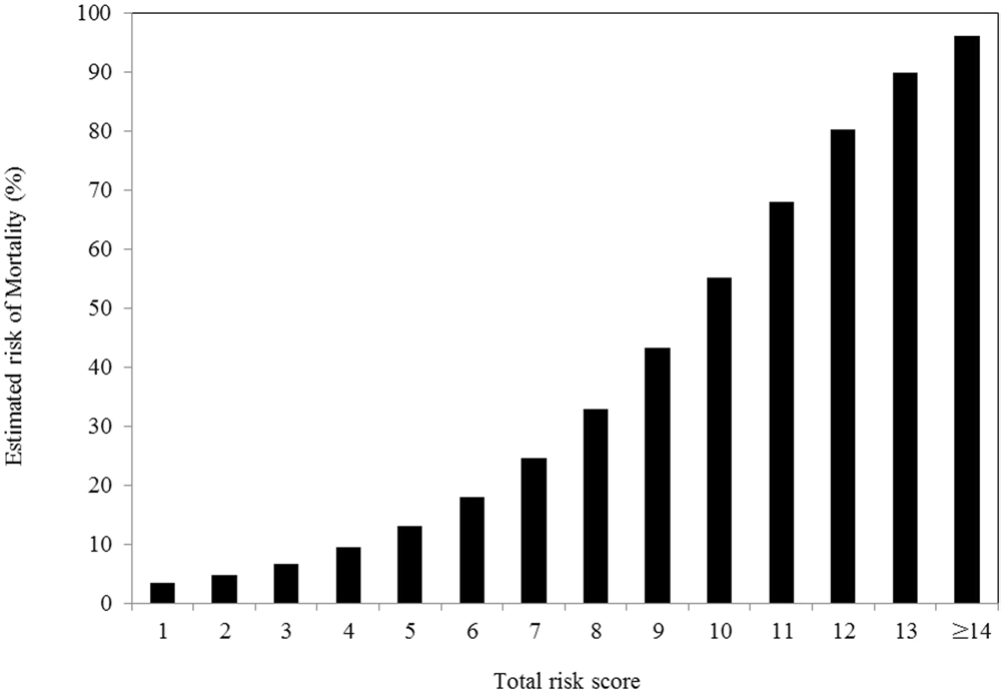
Probability of all-cause mortality in the older subjects with diabetes, according to the point score calculated by the established algorithm. The 5-year probability of mortality was calculated by using the following formula: 1−0.8791^*Exp*{(0.07 × 67 + 0.07 × 5 ×  *point totals*)−6.2926}^.

The risk-score point system is also listed in Table 3. One risk point was added to every five-year increment in age. Two risk points were added to male subjects. Similarly, two risk points were added to those who had fasting blood glucose ≥200 mg/dL, Alb = 3.5–3.9 g/dL, BMI = 18.5–21.9, eGFR < 30 mL/min/1.73 m^2^, WBC ≥ 10,000/µL, or GPT ≥ 120 U/L. Three additional risk points were assigned to those with BMI < 18.5 kg/m^2^ or Hb < 12 g/dL. As shown in Fig. 1, the 5-year estimated mortality rate for those with point totals equal to 5, 10, and 14 were 0.130, 0.552, and 0.962, respectively. The Harrell’s C of the development and of the internal- and external-validation datasets were 0.737, 0.746, and 0.685, respectively.

## Discussion

We established a risk-point system to predict five-year mortality for older adults with diabetes. Physicians and public health workers can apply this handy tool to more accurately identify high-risk groups for individualized counselling or treatment. All 14 parameters used in this risk-point system are essential clinical indicators to monitor older adults with diabetes. In many clinical settings, these data are also collected in annual health check-ups for the elderly. To overcome the capricious nature of diabetes prognosis in older population, this easy-to-use risk-prediction algorithm can serve as a desk reference in daily practice.

Several risk engines have been developed for diabetes patients, targeted at predicting specific disease outcomes such as cardiovascular diseases^17–20^, stroke^21^, and end-stage renal disease^22^. However, few studies have been conducted to assess overall mortality risk for diabetes patients. Wells *et al*. used a type 2 diabetes cohort in the Cleveland Clinic to develop a six-year mortality risk-prediction tool with a C-statistic of 0.752^23^. The uniqueness of this model was the incorporation of anti-diabetes drugs in its development; however, there were two drawbacks in the Well’s risk model. First, the cohort used in the Well’s study was limited only to those “who were prescribed a single one of the four most common types of oral hypoglycemic agents”. By excluding those who used insulin, the Well’s model may not be generalizable to those who had long diabetic duration or severer conditions. Second, there was lack of external validation for their proposed model. Another risk engine, developed by the Hong Kong Diabetes Registry, was to assess five-year mortality risk for Chinese patients with diabetes^13^. The strength of this model was its good discriminatory ability in prediction of five-year mortality risk (C-statistic = 0.845 in the all-cause mortality model). However, the mean age of their study subjects was 57–58 years, indicating that this model may be less accurate in applying to older patients. Moreover, some important indicators of diabetes care, including control of blood pressure and glycemic status, were not included in their model, and the generalizability of this model has not been externally validated yet. In order to supplement the weaknesses of the aforementioned studies, the current study, using common clinical biomarkers to predict mortality risk for older adults with diabetes, surpassed the general geriatric mortality models^10–12^ which seldom had sufficient biomedical profiles applied in their developed models.

Prevention of hypoglycemia is key to optimizing diabetes management for the elderly^7^. Our risk estimate for those with fasting glycemic level <70 mg/dL also concurred with this point. In this study, fasting glucose ≥140 mg/dL was considered as a risk of mortality. Although we adjusted the risk score up to 2 for those with fasting glucose ≥200 mg/dL, due to data availability, our proposed risk point system was not fine-tuned according to the elders’ physical function and health status as recommended in the ADA guidelines for older adults^7^.

For cardiovascular risk prevention, all evidence supports treatment for the elderly with hypertension^24^. Our study showed that uncontrolled high blood pressure (indicated by DBP ≥ 80 mmHg) is a mortality risk, which is lower than the target (90 mmHg) set by the ADA guideline^7^ but supported by the HOT study^25^. Our study also cautioned about potential harm caused by hypotension (indicated by SBP < 110 mmHg), which was usually not precisely emphasized in most diabetes care guidelines but in accord with some RCT and cohort studies. The ACCORD study^9^ did not show protective effects except for stroke prevention for those reducing their SBP < 120 mmHg and a British cohort study^26^ actually demonstrated those with blood pressure < 110/75 mmHg were associated with poor health outcome.

High leukocyte count, long recognized as an indicator of chronic inflammation, has been shown associated with cardiovascular disease^27,28^, cancer, and mortality^29^. We also found high WBC count (≥8,200/μL) was a mortality risk for the elderly. Furthermore, severe chronic inflammation (WBC count ≥ 10,000/μL, risk score = 2), anemia (hemoglobin < 12 g/dL, risk score = 3) and malnutrition (albumin < 3.5 g/dL, risk score = 4; BMI < 18.5 kg/m^2^, risk score = 3), which are often involved in the intricate mechanisms of sarcopenia and frailty^30^, would incur the highest mortality risk for the investigated cohort. In addition to the expected effect of age, our results showed that nutrition status is sensitive and crucial to survivorship in the elderly, whose mortality would be increased by even only a mild decline of hemoglobin (<14 g/dL) and serum albumin level (<4 g/dL). The risk of BMI < 23 kg/m^2^ in the elderly has also been shown in many studies^31^. However, different from some previous research, obesity was not considered as a risk score in the current study due to the fact that our study subjects with BMI > 35 kg/m^2^ were only 1.14%.

If GPT = 40 U/L is considered as the upper limit of normal (ULN)^32^, we found those with elevated GPT but with ULN of less than three would have an aggravated mortality risk (by increasing 1 risk score), whereas the corresponding risk would be doubled for those with GPT > 3 ULN (by increasing 2 risk scores). Elevated GPT, involved in liver dysfunction, was also shown as a dose-response mortality risk in an American community study^33^. Liver enzyme activity should be regularly monitored for the diabetic elderly in Southeast Asia, a region with a high prevalence of viral hepatitis and hepatocellular carcinoma^34^. Some studies have indicated that extremely low GPT — possibly associated with frailty, malnutrition, or hepatic aging process — may also be a mortality risk for the elderly^35^. However, perhaps due to an insufficient sample size for this group (our study subjects with GPT < 5 U/L were 0.05%), this inverse association was not identified in this study.

Our results revealed a U-shaped relationship between mortality and some biochemical indicators — including total cholesterol, uric acid, and eGFR — in the elderly with diabetes. Similar to other studies^36–38^, low cholesterol level (<150 mg/dL), probably related to chronic disease or malnutrition, was identified as a risk factor for all-cause mortality in our study. Moreover, we also recommend that older adults should keep TCHOL below 240 mg/dL to prevent premature mortality. In accordance with other studies^39,40^, a moderate uric acid level was also suggested in this study to balance its dynamic function of antioxidant properties and endothelial integrity for the elderly. Chronic hyperuricemia would stimulate the renin-angiotensin system and inhibit release of endothelial nitric oxide, contributing to vasoconstriction and atherosclerosis, then possibly increase blood pressure and cause renal and cardiovascular disease for the elderly^41,42^. Poor protein intake, hypo-osmolality, underlying diseases and medication may have contributed to the development of lower uric acid level^43^. In line with the traditional guideline^44^ in which eGFR < 60 ml/min/1.73 m^2^ was considered as a cutoff point to define chronic kidney disease, this study confirmed that deterioration of renal function in the elderly was also a mortality risk, especially for those with eGFR < 30 ml/min/1.73 m^2^. Furthermore, our results revealed that extremely high eGFR (>100 ml/min/1.73 m^2^) was a hazardous indicator to older people. The U-shaped relationship between eGFR and mortality has also been observed in an Asian population^45^ and a multinational cohort in the Chronic Kidney Disease Prognosis Consortium^46^.

The handy algorithm demonstrated in this study is the first mortality predication model for the elderly with diabetes. The strengths of this study include its high discriminative ability, its robustness (confirmed by both internal- and external-validation processes), and development of a simple point system that would be easy to use in clinical applications. However, some inherent limitations should be acknowledged. First, because no detailed medical records could be linked to the investigated datasets, we did not include a full list of comorbidity status in our models; however, the comprehensive biochemical profiles used in model development may be able to reveal most clinical information from our study subjects. In addition, because self-reported hypertension and hyperlipidemia were available in the development dataset, we have conducted subgroup analyses for those with hypertension and hyperlipidemia. The estimated risk point system for these two subgroups was shown in the Supplementary Table, in which most of the risk score was similar to that shown in the original model. A moderate weight (+1 or −1) might have to be adjusted for some categories, but the changes have been kept in the same direction, indicating the robustness of our prediction model. Second, health behaviors such as physical activity and drinking pattern were not fully included in our proposed models due to lack of availability; however, we incorporated the most important behavior factor, smoking status, as a covariate in the models. Third, because the Adult Health Screening Program is an ongoing government-sponsored national program, the laboratory data analysis is done by individual medical facilities and cannot be centralized. To ensure the quality of the laboratories, the sponsoring agent, the Health Promotion Administration, accredits the involved laboratories every three years. We thus believe the assay quality of those eligible laboratories is standardized and acceptable. Fourth, it may incur concerns about the moderately lower, although still acceptable, Harrell’s C statistic (0.685) in the external-validation dataset. We acknowledge that it is common to use the data collected in the same period or in the later period to validate the newly established model, but because of the data availability, we had to use the dataset collected in an earlier period for external validation. In addition, our data showed the subjects in the external-validation dataset had significantly better metabolic profiles and lower mortality rates compared to those in the development dataset (Tables 1 and 2). We believe no any datasets in the real world can be found to be completely identical to the development dataset. An acceptable Harrell’s C derived from a distinct dataset may just reveal our model’s robustness and generalizability to Chinese population. Fifth, the available dataset could not allow us to categorize the elderly based on their health status and functional capacity, so the purpose of our calculated risk point system is to estimate mortality risk for the diabetic elders, but we cannot use it to fine-tune the individualized diabetic care plan as suggested by the ADA guideline^7^. Finally, the current results, derived from older Chinese adults with diabetes, may not be directly generalizable to other ethnic groups or younger diabetic patients.

In conclusion, we have developed a simple point system that uses common clinical measures and biochemical profiles in clinical settings to predict five-year mortality risk for the elderly with diabetes. Facing the challenges of a rapid elevation in the aging population and diabetes prevalence, health providers are recommended to take this point system into account to better predict outcomes and to refine treatment strategies for their older diabetic patients.

## Methods

### Study Cohorts

The data used in this study were collected from the Taiwan’s Adult Health Screening Program (AHSP) from 2007 to 2010^47^. The AHSP is a government-sponsored nationwide annual health check-up program for Taiwanese citizens ≥40 years. Information recorded in the AHSP database includes demographics, smoking status, common biochemical measures of blood pressure, lipid, fasting glucose, and kidney and liver function. Diabetes was defined as self-reported or fasting blood glucose ≥126 mg/dL. After excluding those who were younger than 65 years, were not diabetic patients, had missing data, or had an extreme level in the collected biomarkers (the highest and lowest 1%), we selected 245,370 study subjects for further analysis. Every study subject selected in this study was followed up until December 31, 2011 (up to 5 years).

### Internal- and External-Validation Datasets

We used the 10-fold cross-validation method^48^ to randomly allocate the selected AHSP cohort into 10 subsets in which nine subsets were chosen to develop and to fit a prediction model and the remaining subset was used to conduct the internal validation. We repeatedly tested the performance of the combination of the selected development and internal validation subsets until the weighted mean square error (MSE), which was calculated by using the inverse probability of censoring weights (IPCW)^49^, reached minimum among all tests. Finally, the pair with smallest MSE (n = 220,832 for the development subset and n = 24,538 for the internal-validation subset) was selected for further analysis. For the external-validation dataset, we used the MJ Health Screening database (2004–2007), a reliable epidemiological data source in Taiwan^45^, from which we selected 2,093 older people (≥65) with diabetes. The subjects in the external-validation dataset were followed through until December 31, 2008 (up to 5 years).

### Outcome and Definition of Candidate Predictors

The primary outcome investigated in this study was five-year all-cause mortality, which was ascertained by the national death registry. The candidate predictors used for model development were those that were considered as important influencing factors related to diabetic outcomes and health in older people, including gender, age, smoking status, BMI, fasting glucose, systolic and diastolic blood pressure, total cholesterol, triglyceride (TG), hemoglobin, albumin, eGFR, white blood cell count, glutamic pyruvic transaminase (GPT), and uric acid. The investigated predictors were categorized into several levels, as illustrated in Table 2. The same variables and categories were used in the internal and external validation models.

### Statistical Analysis

The categorical variables of baseline demographics and biomarkers were described as frequencies with percentages; and continuous variables were described as means with standard deviations. The points system of this study calculated for both development and validation models were based on the methods proposed by Sullivan *et al*.^50^. First, we classified age and the other continuous variables into the designated categories and then assigned a clinically relevant reference level as the reference category for each investigated covariate. Second, we assessed the regression coefficient for each category of investigated covariates by using the Cox proportional hazards model and computed distance in term of regression unit between different categories and the assigned reference category for each covariate. Third, to construct a point system, we added one point for every five-year increment in age and let 5 multiply the regression coefficient of age as a reference to transform the regression coefficient in each significant category of investigated covariates into a risk point estimate. Finally, the discrimination capabilities of the established regression models were evaluated by Harrell’s C statistics^51^.

We also applied a bootstrapping technique^52^ to evaluate overfitting issue in our prediction model. We generated 200 bootstrap samples with replacement from the original development dataset. The sample size of each bootstrap sample was kept equal to the original dataset (n = 220,832). In each bootstrap model, we evaluated its individual optimism (the difference of the model performance between the bootstrap sample and the original dataset). The average optimism of the Harrell’s C statistics for all 200 bootstrap models due to variable selection and coefficient estimation was 0.0032, indicating there was no sign of overfitting.

Multivariable Cox proportional hazards model was used to determine the independent effects of investigated covariates on all-cause mortality. The survival time of each subject was defined as the time from enrollment to death in all-causes or last follow-up. Relative risks were expressed as hazard ratio (HR) for the comparison between a specific category and the corresponding designated reference level of the investigated covariate. The proportional hazard assumption, the constant HR over time, was evaluated by comparing estimated log–log survival curves for all covariates. We also performed subgroup analyses for those with self-reported hypertension and hyperlipidemia to test robustness of our model. All analyses were performed by using SAS version 9.4 (SAS Institute, Cary, NC, USA) and R software (http://www.r-project.org/). These two-sided tests for all test statistics were regarded as statistical significance if *p* values ≤ 0.05. This study was approved by the institutional review board (IRB) of the National Health Research Institutes. Because the two de-identified databases used in this study were open to the public, the IRB agreed to waive informed consent from the scrambled study subjects. The authors performed the study in accordance with the Declaration of Helsinki.

## Electronic supplementary material

Supplementary Table
