# Supplementary material for: A Point-based Mortality Prediction System for Older Adults with Diabetes
Source: Sci Rep. 2017 Oct 4;7:12652. doi: 10.1038/s41598-017-12751-3 (PMC5627261; doi:10.1038/s41598-017-12751-3)
Supplement: Supplementary file 1 — Supplementary Table [file 41598_2017_12751_MOESM1_ESM.pdf]

A Point-based Mortality Prediction System for Older Adults with Diabetes

Y.K. Chang,[1] L.F. Huang,[2] S.J. Shin,[3,4,5] K.D. Lin,[3,4,5] K. Chong,[6] F.S. Yen,[7] H.Y. Chang,[2] S.Y. Chuang,[2] T.J. Hsieh,[2] C.A. Hsiung,[2] and C.C. Hsu[2,8,9]

[1]Department of Medical Research, Tung's Taichung Metro Harbor Hospital, Taichung, Taiwan. [2]Institute of Population Health Sciences, National Health Research Institutes, Zhunan, Taiwan. [3]Graduate Institute of Medicine, College of Medicine, Kaohsiung Medical University. [4]Ditvision of Endocrinology and Metabolism, Department of Internal Medicine, Kaohsiung Medical University Hospital, Kaohsiung, Taiwan. [5]Department of Internal Medicine, Kaohsiung Municipal Ta-Tung Hospital. [6]Division of Endocrinology and Metabolism, Department of Internal Medicine, Min-Sheng General Hospital, Taoyuan, Taiwan. [7]Dr. Yen's Clinic, Taoyuan, Taiwan. [8]Department of Health Services Administration, China Medical University and Hospital, Taichung, Taiwan. [9]Department of Family Medicine, Min-Sheng General Hospital, Taoyuan, Taiwan.

Correspondence and reprint requests should be addressed to:
Chih-Cheng Hsu, MD, DrPH
Institute of Population Health Sciences, National Health Research Institutes, Zhunan, Taiwan; Department of Health Services Administration, China Medical University and Hospital, Taichung, Taiwan; Department of Family Medicine, Min-Sheng General Hospital, Taoyuan, Taiwan.
35 Keyan Road, Zhunan, Miaoli County 35053, Taiwan
Tel: 886-37-246-166 ext. 36336
Fax: 886-37-586-261
Email: cch@nhri.org.tw

Supplementary Table Risk factors and the point system to predict five-year all-cause mortality for the older diabetic subjects with hypertension (model 1) or hyperlipidemia (model 2)

Risk factor	Model (1) for subjects with hypertension	Model (2) for subjects with hyperlipidemia
Male	2	2
Ever smoker	1	1
BMI (kg/m^2)		
<18.5	3	2
18.5-21.9	2	1
22-23.9	1	1
24-29.9 (reference)	0	0
≥30	0	0
Fasting glucose (mg/dl)		
<70	1	2
70-139 (reference)	0	0
140-159	1	1
160-199	1	1
≥200	2	2
SBP (mmHg)		
<110	1	1
110-119	0	0
120-159 (reference)	0	0
160-169	0	0
≥170	0	1
DBP (mmHg)		
<80 (reference)	0	0
80-89	1	1
≥90	1	1
TCHOL (mg/dl)		
<140	1	1
140-149	1	1
150-239 (reference)	0	0
≥240	1	1
TG (mg/dl)		

<120	0	-1
120-159	0	0
160-199 (reference)	0	0
200-239	0	0
≥240	0	0
Hb (g/dl)		
<12	2	2
12-12.9	1	1
13-13.9	1	1
≥14 (reference)	0	0
Alb (g/dl)		
<3.5	4	4
3.5-3.9	2	2
≥4.0 (reference)	0	0
eGFR (mL/min/1.73m^2)		
≥100	1	0
60-99 (reference)	0	0
45-59	1	1
30-44	1	1
<30	2	2
WBC (/μL)		
<8,200 (reference)	0	0
8,200-9,999	1	1
≥10,000	2	2
GPT (U/L)		
<40 (reference)	0	0
40-79	0	0
80-119	1	1
≥120	2	0
UA (mg/dl)		
<3.5	1	1
3.5-6.9 (reference)	0	0
7-8.9	0	1
≥9	1	1
